# Featured Species-Specific Loops Are Found in the Crystal Structure of *Mhp* Eno, a Cell Surface Adhesin From *Mycoplasma hyopneumoniae*

**DOI:** 10.3389/fcimb.2019.00209

**Published:** 2019-06-13

**Authors:** Rong Chen, Yanfei Yu, Zhixin Feng, Rong Gan, Xing Xie, Zhenzhen Zhang, Qingyun Xie, Weiwu Wang, Tingting Ran, Wei Zhang, Qiyan Xiong, Guoqing Shao

**Affiliations:** ^1^Key Laboratory of Veterinary Biological Engineering and Technology of Ministry of Agriculture, National Center for Engineering Research of Veterinary Bioproducts, Institute of Veterinary Medicine, Jiangsu Academy of Agricultural Sciences, Nanjing, China; ^2^College of Veterinary Medicine, Nanjing Agricultural University, Nanjing, China; ^3^Key Laboratory of Agricultural and Environmental Microbiology, College of Life Sciences, Ministry of Agriculture, Nanjing Agricultural University, Nanjing, China; ^4^Key Lab of Animal Bacteriology of Ministry of Agriculture, OIE Reference Lab for Swine Streptococcosis, College of Veterinary Medicine, Nanjing Agricultural University, Nanjing, China; ^5^Institute of Life Sciences, Jiangsu University, Zhenjiang, China

**Keywords:** enolase, adhesin, *Mycoplasma hyopneumoniae*, plasminogen, structure

## Abstract

Enolase is an evolutionarily conserved enzyme involved in the processes of glycolysis and gluconeogenesis. *Mycoplasma hyopneumoniae* belongs to *Mycoplasma*, whose species are wall-less and among the smallest self-replicating bacteria, and is an important colonizing respiratory pathogen in the pig industry worldwide. *Mycoplasma hyopneumoniae* enolase (*Mhp* Eno) expression is significantly increased after infection and was previously found to be a virulence factor candidate. Our studies show that *Mhp* Eno is a cell surface-localized protein that can adhere to swine tracheal epithelial cells (STECs). Adhesion to STECs can be specifically inhibited by an *Mhp* Eno antibody. *Mhp* Eno can recognize and interact with plasminogen with high affinity. Here, the first crystal structure of the mycoplasmal enolase from *Mycoplasma hyopneumoniae* was determined. The structure showed unique features of *Mhp* Eno in the S3/H1, H6/S6, H7/H8, and H13 regions. All of these regions were longer than those of other enolases and were exposed on the *Mhp* Eno surface, making them accessible to host molecules. These results show that *Mhp* Eno has specific structural characteristics and acts as a multifunctional adhesin on the *Mycoplasma hyopneumoniae* cell surface.

## Introduction

Mycoplasma species are wall-less bacteria with minute genomes that are believed to have evolved by degenerative evolution from Firmicutes (Sladek, 1986; Razin et al., 1998). As the smallest self-replicating organisms, synthetic genome projects for Mycoplasma species have led to an unprecedented understanding of the minimal constituents of the genes enabling life (Glass et al., 2006; Gibson et al., 2010; Hutchison et al., 2016). Mycoplasmas are widespread in the natural world as important parasites of humans, mammals, reptiles, fish, arthropods, and plants (Razin et al., 1998). Among them, *Mycoplasma hyopneumoniae* (*M. hyopneumoniae, Mhp*) is the causative agent of enzootic pneumonia (EP), which is a chronic respiratory disease in pigs and causes significant economic loss in pig production globally (Maes et al., 2017). Intermittent and dry cough, labored breathing, anorexia, lethargy and emaciation are the characterized clinical signs of *M. hyopneumoniae* infection (Maes et al., 1996; Sibila et al., 2009). Lung lesions, composed of purple to gray consolidated areas in the apical, middle and cranial lung lobes in the infected animals, are typical pathological features of the disease (Garcia-Morante et al., 2016). In addition, affected pigs easily suffer from infections by other respiratory pathogens, including viruses, bacteria, and parasites, which can increase the severity of the illness (Li et al., 2015a; Kureljusic et al., 2016).

*M. hyopneumoniae* is mainly found on the mucosal surface of swine trachea, bronchi and bronchioles (Blanchard et al., 1992), inducing ciliostasis and loss of cilia (DeBey and Ross, 1994). The first step of infection caused by *M. hyopneumoniae* is due to successful adhesion to the cilia of the epithelium of the swine respiratory tract (DeBey and Ross, 1994). The P97/P102 paralogue families are recognized as the primary adhesins of *M. hyopneumoniae* (Adams et al., 2005). Repeat regions 1 and 2 are the most important areas of P97 for adhesion (Hsu and Minion, 1998; Minion et al., 2000). However, the host cell-binding region can also be expanded by posttranslational processing, which was recently confirmed by several proteomic studies of *M. hyopneumoniae* (Tacchi et al., 2016). P159 (Raymond et al., 2013), amino peptidases MHJ_0461 and MHJ_0125 (Robinson et al., 2013; Jarocki et al., 2015), EF-Tu (Yu et al., 2018a), and some other moonlighting proteins are newly determined adhesins. The genomics and comparative genomics of several strains of field and attenuated *M. hyopneumoniae* also provide clues to the pathogenicity of *M. hyopneumoniae* (Liu et al., 2013; Siqueira et al., 2013). Glycosaminoglycans, plasminogen, fibronectin, actin and some other molecules are the target molecules of *M. hyopneumoniae* adhesion in the swine respiratory tract epithelium (Jenkins et al., 2006; Deutscher et al., 2010; Jarocki et al., 2015; Raymond et al., 2018). However, few virulence factors of *M. hyopneumoniae* have been identified. Comparative proteomic analyses performed previously in our lab showed that six proteins, including enolase, had relatively higher expression in *M. hyopneumoniae*-infected swine tracheal epithelial cells (STECs) (Yu et al., 2018a).

Enolase (EC 4.2.1.11) is a conserved metalloenzyme present in almost all organisms of the phylogenetic tree (Ehinger et al., 2004; Kang et al., 2008; Sun et al., 2017) that plays a role as a phosphopyruvate hydratase in the reversible chemical reaction of 2-phosphoglycerate (2-PG) to phosphoenolpyruvate (PEP) in the glycolysis pathway (Wold and Ballou, 1957a,b; Reed et al., 1996). The localization of enolase to the cell surface, cytoplasm, and nuclei has been reported in prokaryotic and eukaryotic cells. Due to its short signaling sequence, the transport mechanism remains unknown (Seweryn et al., 2007). Surface enolase is recognized as a plasminogen receptor in several pathogens and is thought to be involved in extracellular matrix degradation and host cell invasion (Ehinger et al., 2004; Díaz-Ramos et al., 2012). Enolase is also reported to participate in the processing of the RNase E degradosome in *Bacillus subtilis* and *Escherichia coli* (Kühnel and Luisi, 2001; Newman et al., 2012; Bruce et al., 2018). The assembly of monomer enolase varies among species and is present either as a dimer or octamer but seldom both (Brown et al., 1998; Lu et al., 2012; Wu et al., 2015). To date, the crystal structures of enolase from various species, including human, flies, and bacteria, have been determined (Kang et al., 2008; Lu et al., 2012; Sun et al., 2017). However, the structure of the enolase of *Mycoplasma*, which is found in an independent evolutionary branch of bacteria, remains unknown.

Here, we reported the first three-dimensional (3D) structure of *Mycoplasma* enolase (*Mhp* Eno, short for *Mycoplasma hyopneumoniae* Enolase). The structure showed a unique *Mycoplasma* enolase with characteristic long loops in the S3/H1, H6/S6, H7/H8, and H13 regions. As a virulence factor candidate found in our previous studies, *Mhp* Eno showed a surface localization on *M. hyopneumoniae* cells as determined by flow cytometry and electron microscopy. *Mhp* Eno specifically adhered to STECs and recognized the host plasminogen. All of these results show the unique features of *Mhp* Eno.

## Materials and Methods

### Ethics Statement

All animal experiments were approved by the Committee on the Ethics of Animal Experiments and performed at Jiangsu Academy of Agricultural Sciences (License No. SYXK (Su) 2015-0019). The animal experiments were conducted following the guidelines of the Animal Regulations of Jiangsu Province (Government Decree No. 45) in accordance with international law, and the animals did not suffer excessively.

### Bacterial Strains and Culture

All *M. hyopneumoniae* strains were maintained and cultured in our lab. *M. hyopneumoniae* strain 168 is a field strain initially isolated in Gansu Province, China (Liu et al., 2013). The pathogenic strains NJ and WX were isolated in Nanjing City and Wuxi City, respectively. KM2 cell-free liquid medium (a modified Friis medium) containing 20% (v/v) swine serum was used for *M. hyopneumoniae* cultures.

### Protein Expression and Purification

*Mhp Eno* (MHP168_271) was synthesized by GenScript Biotech Corp. (Nanjing) and expressed using the pET21a vector in the BL21(DE3) *E. coli* strain. The bacterial cells were grown in LB medium at 37°C until the OD600 reached approximately 0.8. A final concentration of 0.25 mM IPTG was added to the culture for protein expression at 18°C. The cells were collected by centrifugation and resuspended in buffer A [30 mM Tris–HCl (pH 8.0), 300 mM NaCl, and 20 mM imidazole]. The cells were then lysed by sonication and centrifuged at 100,000 × g for 30 min. The soluble fraction was incubated with Ni Sepharose 6 FF resin (GE Healthcare) for 1 h at 4°C. *Mhp* Eno proteins were eluted in buffer A by adding 200 mM imidazole and concentrated by ultrafiltration using Centricons (Amicon). The proteins were dialyzed in buffer B [30 mM Tris–HCl (pH 8.0) and 300 mM NaCl]. Further purification was performed using a Superdex 200 Increase 10/300 GL column (GE Healthcare).

### Crystallization and Structural Analyses

Size exclusion-purified *Mhp* Eno was concentrated and diluted to 10 mg/mL and 15 mg/mL for crystal screening. The crystallization experiment was performed using the sitting-drop vapor diffusion method at 291 K. Crystals of *Mhp* Eno grew in 0.056 M sodium phosphate monobasic monohydrate and 1.344 M potassium phosphate dibasic at pH 8.4 after 20 days. The *Mhp* Eno crystal refracted to 2.3 Å at beamline BL19U1 at the Shanghai Synchrotron Radiation Facility (SSRF). X-ray diffraction data were merged, integrated and scaled using HKL3000 software. The structure of *Mhp* Eno was solved by molecular replacement using the enolase molecule from *Bacillus subtilis* (Protein Data Bank [PDB] ID: 4A3R) as a reference model with Phaser in the CCP4 program suite (Hough and Wilson, 2018). REFMAC5 (Kovalevskiy et al., 2018) and COOT (Emsley and Cowtan, 2004) were used for initial restrained rigid-body refinement and manual model building, respectively. Further refinement was performed with Phenix (DiMaio et al., 2013). The stereochemical quality of the final model was further evaluated with the program PROCHECK. Structural analysis was performed using PyMOL software and the CCP4 program. Dali server was used for the structural similarity comparison.

### Preparation of an Anti-*Mhp* Eno Polyclonal Antibody

A polyclonal antibody was raised against *Mhp* Eno by subcutaneously immunizing 1-month-old New Zealand white rabbits. Each rabbit was immunized three times with 1 mg of *Mhp* Eno emulsified in Freund's adjuvant (Sigma, USA) at 2-week intervals. Sera were collected 1 week after the third immunization.

### Flow Cytometry

Flow cytometry was performed according to a previous study (Yu et al., 2018a). *M. hyopneumoniae* strains [1 × 10^8^ color change units (CCUs)/mL for each) were incubated with anti-*Mhp* Eno serum at a 1:100 dilution (1:100 diluted preimmune serum was used as a negative control, and phosphate-buffered saline (PBS) was used as a blank control). *M. hyopneumoniae* cells were then stained with fluorescein isothiocyanate (FITC)-conjugated anti-IgG, and the fluorescence intensity was measured using a BD Accuri C6 flow cytometer. The assay was performed in triplicate, and data were analyzed with Student's *t*-tests using SPSS 20.0. For all tests, *p* ≤ 0.05 was considered statistically significant.

### Immune Electron Microscopy

*M. hyopneumoniae* strains were grown to mid-log phase and harvested by centrifugation at 6,600 × g at 4°C. A bacterial suspension containing 1 × 10^8^ CCUs was washed thrice with PBS and resuspended in 50 μL of PBS. Twenty microliters of each sample was added to 200-mesh formvar-coated nickel grids and allowed to stand for 5 min. The grids were subsequently fixed with 2% paraformaldehyde in PBS (pH 7.4) for 5 min at room temperature, blocked for 1 h using 1% normal rabbit serum and 1% BSA/PBS and incubated with 1:10 polyclonal rabbit antibody to *Mhp* Eno in the above-mentioned blocking buffer for 1 h (1:10 diluted preimmune serum was used as a negative control, and PBS was used as a blank control). The samples were washed five times for 5 min in blocking buffer and then incubated with secondary gold-conjugated antibodies at 1:20 (goat anti-rabbit IgG, 10-nm gold particles) for another 1 h. Before fixation for 5 min in 2% paraformaldehyde/PBS, the samples were washed five times with PBS for 5 min. The grids were then washed in distilled water eight times and stained for 15 s with 1% phosphotungstic acid (pH 6.5). The samples were observed with a Tecnai high-field transmission electron microscope after drying with an infrared lamp.

### Indirect Immunofluorescence Assays

STECs were maintained and cultured in our lab (Yu et al., 2018a). After incubation with 100 μg of purified *Mhp* Eno at 37°C for 1 h, the cells were washed three times with PBS and incubated with the anti-*Mhp* Eno antibody at a 1:1,000 dilution and then with tetramethylrhodamine isothiocyanate (TRITC)-tagged anti-IgG (Proteintech, 1:500 dilution). Finally, the cell nuclei were stained with 6-diamidino-2-phenylindole (DAPI). The fluorescence was detected using a fluorescence microscope (Zeiss, Germany). BSA and its antibody were used instead of *Mhp* Eno and the anti-*Mhp* Eno antibody as negative controls.

### Inhibition of Adherence Test

*M. hyopneumoniae* cells (1 × 10^7^ CCUs/mL) were washed three times with PBS and preincubated with the polyclonal antibody against *Mhp* Eno or preimmune sera (1:20 dilution) at 37°C for 30 min. The bacteria suspended in RPMI-1640 medium were added to 24-well cell plates containing confluent STECs, and the plates were centrifuged at 800 × g for 10 min and incubated at 4°C for 2 hours. Following incubation, the wells were washed three times with PBS to remove unbound *M. hyopneumoniae* cells. The cells in the wells were treated with lysis buffer containing 0.1% trypsin and 0.025% (v/v) Triton X-100 and then subjected to bacterial genome extraction and real-time PCR for bacterial counting according to a previous method (Yu et al., 2018a). The assay was performed in triplicate, and the data were analyzed by Student's *t*-tests using SPSS 20.0.

### Far-Western Blot (far-WB) Analysis

A 20-μg sample of *Mhp* Eno was separated by SDS-PAGE and transferred to a PVDF membrane (Li et al., 2015b). After blocking with 5% (w/v) skimmed milk, the membrane was incubated with 5 μg/mL fibronectin (Roche), complement factor H (Hycult Biotech) or plasminogen (Sigma) and then with anti-fibronectin, anti-factor H or anti-plasminogen antibody (Abcam; 1 μg/mL) as the primary antibody and horseradish peroxidase (HRP)-conjugated anti-IgG (Boster; 1:5,000 dilution) as the secondary antibody. Finally, the membrane was developed with electrochemiluminescence (ECL) substrate using a ChemiDoc XRS+ system (Bio-Rad). BSA was used instead of *Mhp* Eno as a negative control, and the polyclonal antibody against *Mhp* Eno was used as a positive control.

### Surface Plasmon Resonance (SPR) Analysis

SPR was performed by a BIAcore X100 Plus instrument (GE Healthcare). Plasminogen, fibronectin and factor H were separately diluted to 50 μg/mL and covalently linked to the CM5 sensor chip as a ligand using an amine coupling kit (Biacore AB). The immobilization of soluble plasminogen, fibronectin, and factor H generated resonance units (RU) of ~2,000. The binding kinetics were measured with increasing concentrations (0–4,000 nmol/L) of the analytes (*Mhp* Eno) in running buffer (HBS-EP) consisting of 10 mM HEPES, 150 mM NaCl, 3 mM EDTA, and 0.05% (v/v) surfactant P20 (Biacore AB) at a flow rate of 30 μL/min for 180 s over immobilized *Mhp* Eno at 20°C. The dissociation phase was monitored for 1000 s by allowing buffer to flow over the chip. The association kinetics were analyzed manually using Biacore X100 Control software.

### Enzymatic Activity Assays

Spectrophotometric assays were performed to detect the catalytic reaction at a temperature of 25°C. The reaction was followed by an increase in PEP, which exhibited UV absorbance at a wavelength of 240 nm. To determine the velocity of the enzyme-catalyzed reaction, measurements were performed at 0.5-min intervals for 5.5 min. The concentrations of 2-PGA (Sigma) and *Mhp* Eno used in the assay were 1 mmol/L and 1 μg/mL, respectively. The reaction buffer containing 50 mmol/L Tris–HCl (pH 8.0), 100 mmol/L NaCl, and 1.5 mmol/L MgSO4. 2-PGA was added immediately, and the mixture was mixed well before measurement. Enzymatic activity assays for yeast enolase (Sigma, the positive control) and BSA (the negative control) were performed using the same protocol. The specific activity was calculated using the following equation:

$$\begin{array}{l} \text{U}/mg=\frac{\Delta \text{OD}}{t\times \varepsilon \times l\times m}\times V\times 10^{6} \end{array}$$

### Protein Data Bank Accession Number

The coordinates and structural factors generated in this study were submitted to the Protein Data Bank (https://deposit-pdbj.wwpdb.org/deposition/) under the following accession number: 6J36.

## Results

### Surface Localization of Enolase on *Mycoplasma hyopneumoniae* Cells

**Figure 1:**
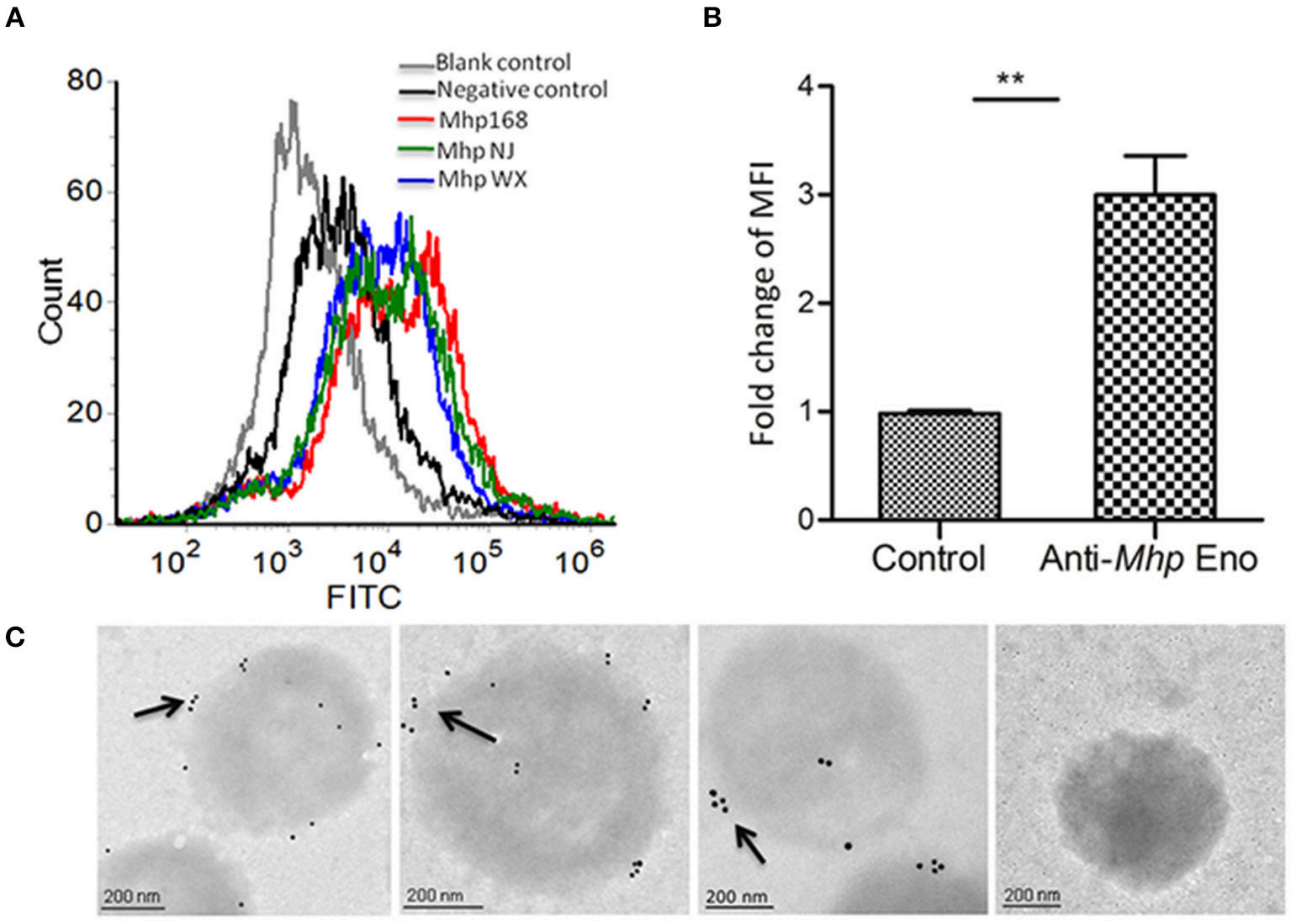
Detection of *Mhp* Eno on the surface of *M. hyopneumoniae* by flow cytometry and immune electron microscopy. **(A)** Blank control, *M. hyopneumoniae* strain 168 treated with PBS; negative control, *M. hyopneumoniae* strain 168 treated with preimmune serum; *M. hyopneumoniae* strains 168, NJ, and WX treated with anti-*Mhp* Eno serum. **(B)** The MFI level of *M. hyopneumoniae* incubated with anti-*Mhp* Eno sera is expressed as the percentage of that found for the corresponding strain incubated with preimmune sera. The asterisks above the charts indicate statistically significant differences. **(C)** From left to right, the first three *M. hyopneumoniae* strains, 168, NJ and WX, were treated with anti-*Mhp* Eno serum and secondary gold-conjugated antibodies. The last column indicates the *M. hyopneumoniae* strain treated with preimmune serum and secondary gold-conjugated antibodies.

Our previous studies showed that *Mhp* Eno is a candidate virulence factor of *M. hyopneumoniae* (Yu et al., 2018a). Enolases of bacterial pathogens often have a surface localization, which contributes to mucosal surface colonization and host tissue invasion (Bergmann et al., 2001; Ehinger et al., 2004; Agarwal et al., 2008). To investigate the cell surface localization of enolase on *M. hyopneumoniae*, two approaches were used. A flow cytometry analysis showed that the mean fluorescence intensity (MFI) of *M. hyopneumoniae* strains 168, NJ and WX treated with anti-*Mhp* Eno serum was 3-fold higher than that of strains treated with preimmune serum (Figures 1A,B), which indicated that the surface of *Mhp* Eno was accessible to the *Mhp* Eno-specific antibody.

In addition, immune electron microscopy was further applied to study the surface localization of *Mhp* Eno. The results revealed that *Mhp* Eno was localized and restricted around the outer region of the bacteria cell for all three strains of *M. hyopneumoniae* used in this study, whereas the gold particles were not visible in the negative control group treated with preimmune sera (Figure 1C and Supplementary Figure 1). All of these results revealed the surface-accessible localization of *Mhp* Eno. We did not confirm the distribution of *Mhp* Eno in the cytoplasm of *M. hyopneumoniae*, but it is assumed that *Mhp* Eno also exists in the cytoplasm. First, enolase is a highly conserved enzyme in the glycolysis pathway. Second, the enzymatic activity of *Mhp* Eno is similar to that of natural yeast enolase (Supplementary Figure 2). Third, it is common for enolase to show multiple localizations at the cell surface, cytoplasm and nuclei (Kang et al., 2008).

### Adherence of *Mhp* Eno to STECs

**Figure 2:**
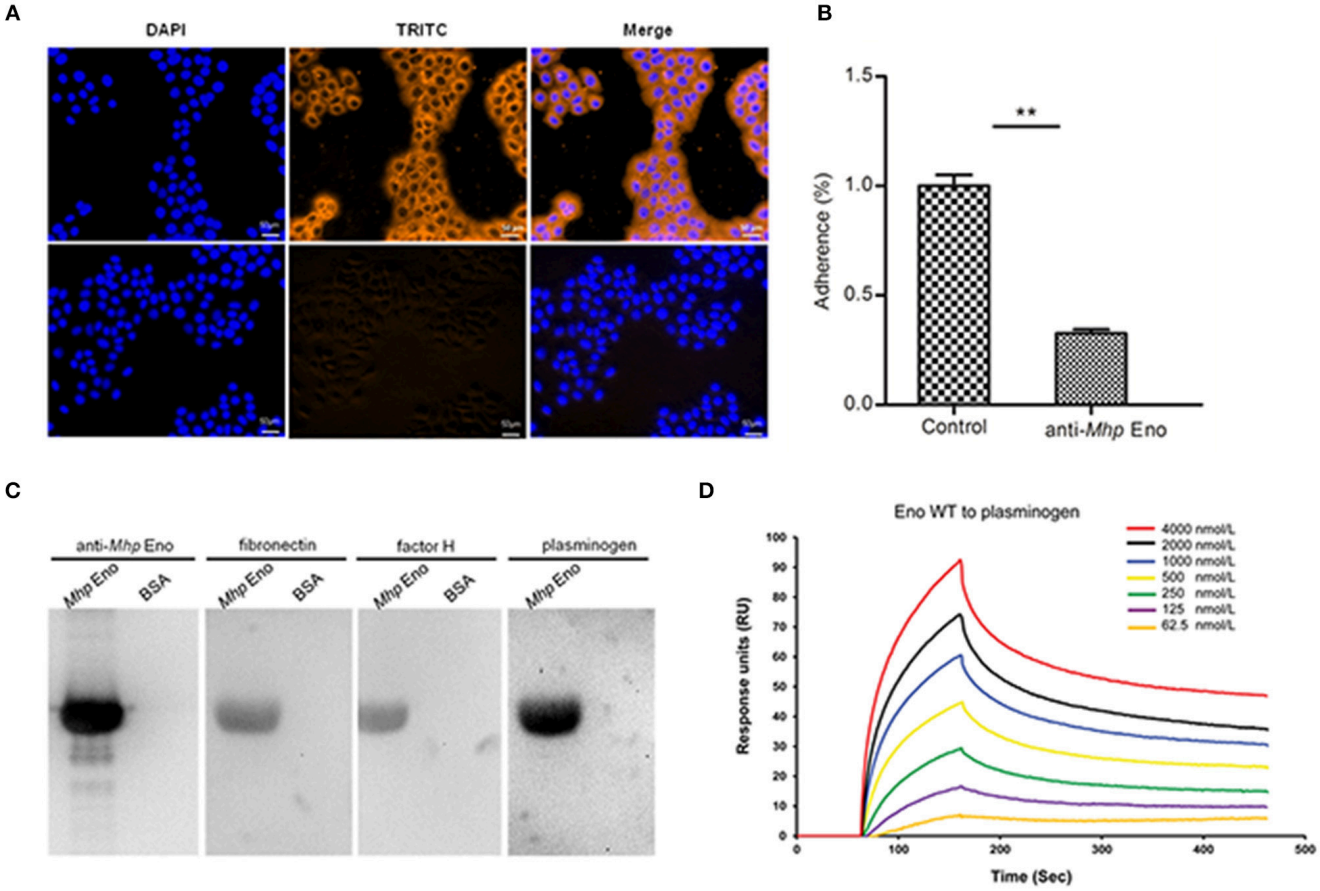
Role of *Mhp* Eno in the adhesion of *M. hyopneumoniae* to STECs. **(A)** Blue indicates STEC nuclei. The color orange in the first row indicates the adherence of *Mhp* Eno to STEC membranes, and the second row indicates the adherence of BSA (negative control) to STEC membranes. The white line indicates the scale. **(B)** Adhesion rate = (number of bacteria recovered from cells incubated with the anti-*Mhp* Eno sera / number of bacteria recovered in the group incubated with the preimmune sera) × 100. The data are expressed as the means ± SDs of at least three experiments with samples performed in triplicate. **(C)** Analysis of the interaction of *Mhp* Eno with fibronectin, factor H and plasminogen by far-WB. First lane: PVDF membrane with transferred *Mhp* Eno protein incubated with anti-*Mhp* Eno antibody as a positive control; second lane: PVDF membrane with transferred BSA (negative control) incubated with anti-*Mhp* Eno antibody; third lane: PVDF membrane with transferred *Mhp* Eno protein incubated with fibronectin and anti-fibronectin antibody; fourth lane: PVDF membrane with transferred BSA (negative control) incubated with fibronectin and anti-fibronectin antibody; fifth and sixth lanes: PVDF membrane with transferred *Mhp* Eno protein and BSA incubated with factor H and anti-factor H antibody; and seventh and eighth lanes: PVDF membrane with transferred *Mhp* Eno protein and BSA incubated with plasminogen and the anti-plasminogen antibody. The protein bands were visualized using the ECL substrate. **(D)** Gradient concentrations of *Mhp* Eno enolase ranging from 62.5 to 4,000 nmol/L flow through immobilized plasminogen in an SPR assay. The protein concentrations were consistent with the color lines. RU, resonance units.

To explore the potential relationship between the surface localization of *Mhp* Eno and its function, the adhesive ability of *Mhp* Eno to STECs was studied by indirect immunofluorescence. Significant fluorescence was observed on the cell surface of STECs incubated with *Mhp* Eno. However, no specific fluorescence was detected around the DAPI-stained cell nuclei in the negative controls (Figure 2A). These results provided direct evidence showing that *Mhp* Eno binds specifically to the cell membranes of STECs.

Antibody inhibition assays were further performed to determine the contribution of surface-localized *Mhp* Eno to the adhesion function in *M. hyopneumoniae* strain 168. A polyclonal antibody against *Mhp* Eno was found to decrease the adherence of *M. hyopneumoniae* to STECs relative to that of the control group treated with preimmune sera (Figure 2B). The level of adherence inhibition is shown as percentages compared with the adherence of *M. hyopneumoniae* in the absence of antibody. Incubation with anti-*Mhp* Eno antibody resulted in a 68% (*p* < 0.05) reduction in the adherence efficiency of *M. hyopneumoniae* to STECs. This result confirmed that *Mhp* Eno plays an indispensable role in the adherence of *M. hyopneumoniae* to host cells.

### *Mhp* Eno Exhibits High Affinity for Plasminogen-Binding Activities

To determine which components of STECs interact with *Mhp* Eno, the interactions between *Mhp* Eno and fibronectin, factor H and plasminogen were examined by far-WB analysis. Corresponding bands were observed in the reactions of *Mhp* Eno to the anti-*Mhp* Eno antibody (positive control) and to fibronectin, factor H and plasminogen, whereas no specific reaction was observed in the negative control. The analysis indicated that *Mhp* Eno specifically binds to fibronectin, factor H and plasminogen (Figure 2C). The real-time interactions between *Mhp* Eno and fibronectin, factor H and plasminogen were further investigated using SPR. However, no interactions were detected between *Mhp* Eno and fibronectin or factor H, indicating weak interactions. *Mhp* Eno was found to bind to plasminogen in a dose-dependent manner with an equilibrium dissociation constant (*K*_D_) value of 293.6 nmol. Only 62.5 nmol/L *Mhp* Eno yielded notable reflection signals (Figure 2D). All of these results indicated that *Mhp* Eno has multiple adhesive functions, and its highest affinity was to plasminogen.

### Unique Structure of *Mhp* Eno

**Figure 3:**
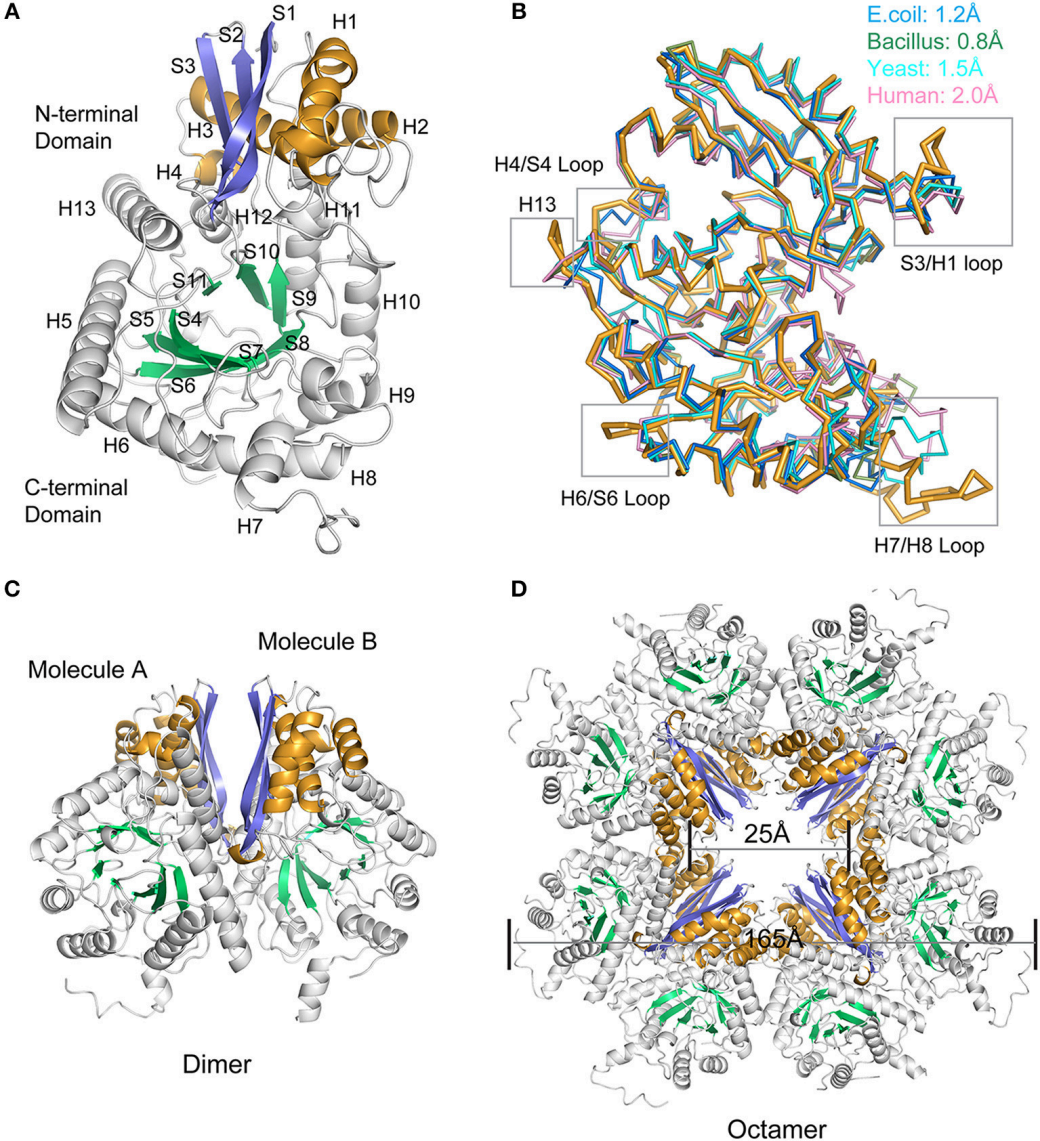
Overall structure of *Mhp* Eno. **(A)** Structure of the protomer *Mhp* Eno. The β-strands and α-helices are sequentially numbered; “S” stands for β-strand, and “H” stands for α-helix. The β strands of the N-terminal domain and C-terminal domain are indicated in slate (S1-S3) and green cyan (S4-S10) colors, respectively. The α-helices of the N-terminal domain and C-terminal domain are indicated in bright orange (H1-H4) and white (H5-H13), respectively. **(B)** Structural comparisons between *Mhp* Eno and enolases from human (pink, PDB ID: 3B97), yeast (cyan, PDB ID: 3ENL), *E. coli* (blue, PDB ID: 1E9I), and *Bacillus subtilis* (green, PDB ID: 4A3R). All structures are shown in a ribbon format. *Mhp* Eno is shown in bright orange. The structure deviations are indicated at the top. The regions of *Mhp* Eno that show noticeable differences are marked by gray boxes. **(C)** Overall structure of the *Mhp* Eno dimer unit. **(D)** Overall structure of the *Mhp* Eno octamer. The diameters of *Mhp* Eno and the tunnel are indicated.

The oligomeric state of enolase, particularly enolases from prokaryotes, is always a topic of debate. Here, in *Mycoplasma hyopneumoniae*, an octamer form was determined for *Mhp* Eno (Figure 3A). The crystal structure was determined at a resolution of 2.3 Å (Figure 3 and Table 1). Two identical *Mhp* Eno monomers formed a “heart”-like or “butterfly”-like dimer (Figure 3C). Four dimers were then packed together to construct a ring-shaped octamer with a small tunnel in the center. Both octamer interfaces and dimer interfaces were observed in *Mhp* Eno. These structures were similar to those of other solved enolase structures (Supplementary Tables 1, 2). The diameters of the octamer *Mhp* Eno disc and the center tunnel were ~165 Å and ~25 Å, respectively (Figure 3D). Similar to enolases from other species, the overall structure of monomer *Mhp* Eno could be divided into an N-terminal domain and a C-terminal TIM-barrel domain. The N-terminal domain (residues 1–139) comprised an antiparallel three-stranded β-sheet (S1–S3) followed by four α-helices (H1– H4). The C-terminal domain (residues 140–452) was relatively larger and consisted of eight β-strands (S4–S11) and nine α-helices (H5–H13). Compared with other enolases, *Mhp* Eno had an additional α-helix, which was located on the site corresponding to the S6-H7 loop of other enolase structures, named H7 in *Mhp* Eno. The C-terminal domain topology consisted of a β_2_α_2_βα_2_(βα)_5_ pattern, which differed from either the traditional TIM-barrel domain or those of other enolases (Figure 3A).

**Table 1:** Data collection and refinement statistics.

	***Mhp* Eno**
**Data collection**	
Beamline	SSRF BL19U1
Space group	*I*4
Cell Dimensions	
*a, b, c* (Å)	193.154, 193.154, 63.943
α, β, γ (°)	90.0, 90.0, 90.0
Wavelength (Å)	0.97892
Resolution (Å)	48.29–2.30 (2.44–2.30)
Total no. of reflections	241,925
*R*_merge_ (%)	0.108 (0.597)
*I*/σ*I*	14.2 (3.07)
Completeness (%)	99.8 (99.8)
Redundancy	4.6 (4.7)
**Refinement**	
Resolution (Å)	48.29–2.30
No. reflections	52,557
*R*_work_/*R*_free_ (%)	0.1865/0.2257
No. atoms	7,101
Protein	7,000
Water	57
Average *B*-factors (Å^2^)	
Protein	31.3
Water	24.1
Rmsd Values	
Bond lengths (Å)	0.008
Bond angles (°)	0.886
Ramachandran plot (%)	
Total favored	96.44%
Total allowed	3.11%
Outliers	0.44%
Coordinate error (Å)	0.29

* The values in parentheses are for the highest-resolution shell.

R_merge_ = ∑ | I−〈I〉| /∑I, where I is the integrated intensity of a given reflection.

R = ∑ || F_obs_ | – | F_calc_ | |/∑_hkl_ | F_obs_ |.

R_free_ was calculated using 5% of the data omitted from the refinement.

*I /σI = average (I /σI)*.

**Figure 4:**
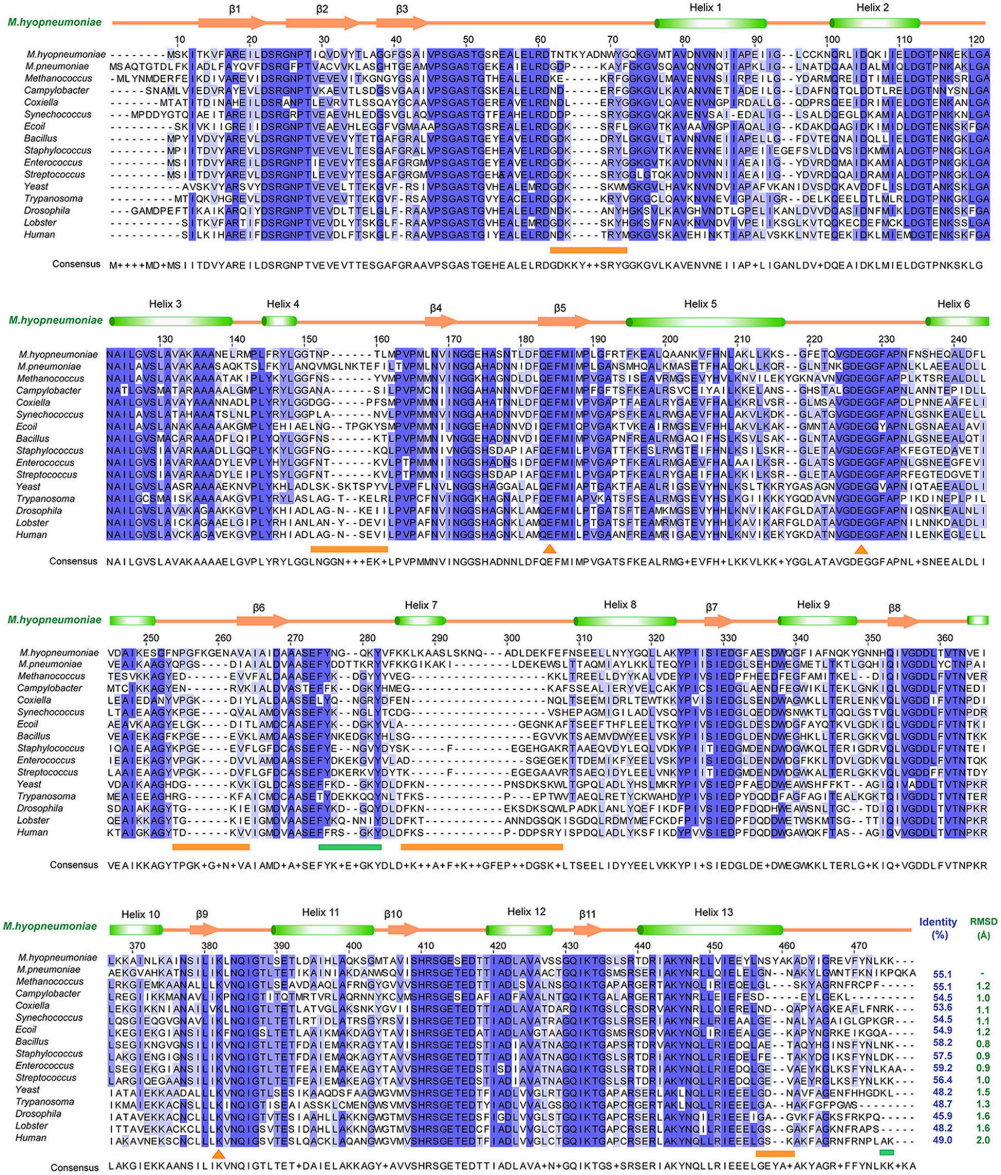
Structural and sequence alignments between *Mhp* Eno and other enolases. The sequence identities and structural deviations are shown at the end of each sequence. The second structure of *Mhp* Eno is above the alignment. The regions that show notable differences are indicated with orange lines at the bottom of the alignment. The plasminogen-binding regions are marked by green lines at the bottom of the sequence. The sequences or structures are from *M. pneumoniae* (*Mycoplasma pneumoniae*, GenBank ID: WP_010874963.1), Methanococcus (*Methanococcus jannaschii*, PDB ID: 2PA6), Campylobacter (*Campylobacter jejuni*, PDB ID: 3QN3), Coxiella (*Coxiella burnetii*, PDB ID: 3TQP), Synechococcus (*Synechococcus elongatus*, PDB ID: 4ROP), *E. coli* (PDB ID: 1E9L), Bacillus (*Bacillus subtilis*, PDB ID: 4A3R), Staphylococcus (*Staphylococcus aureus*, PDB ID: 5BOF), Enterococcus (*Enterococcus hirae*, PDB ID: 1LYX), Streptococcus (*Streptococcus pneumonia*, PDB ID: 1W6T), Yeast (PDB ID: 3ENL), Trypanosoma (*Trypanosoma brucei*, PDB ID: 1OEP), Drosophila (PDB ID: 3WRO), Lobster (PDB ID: 1PDZ), and Human (PDB ID: 3B97).

**Figure 5:**
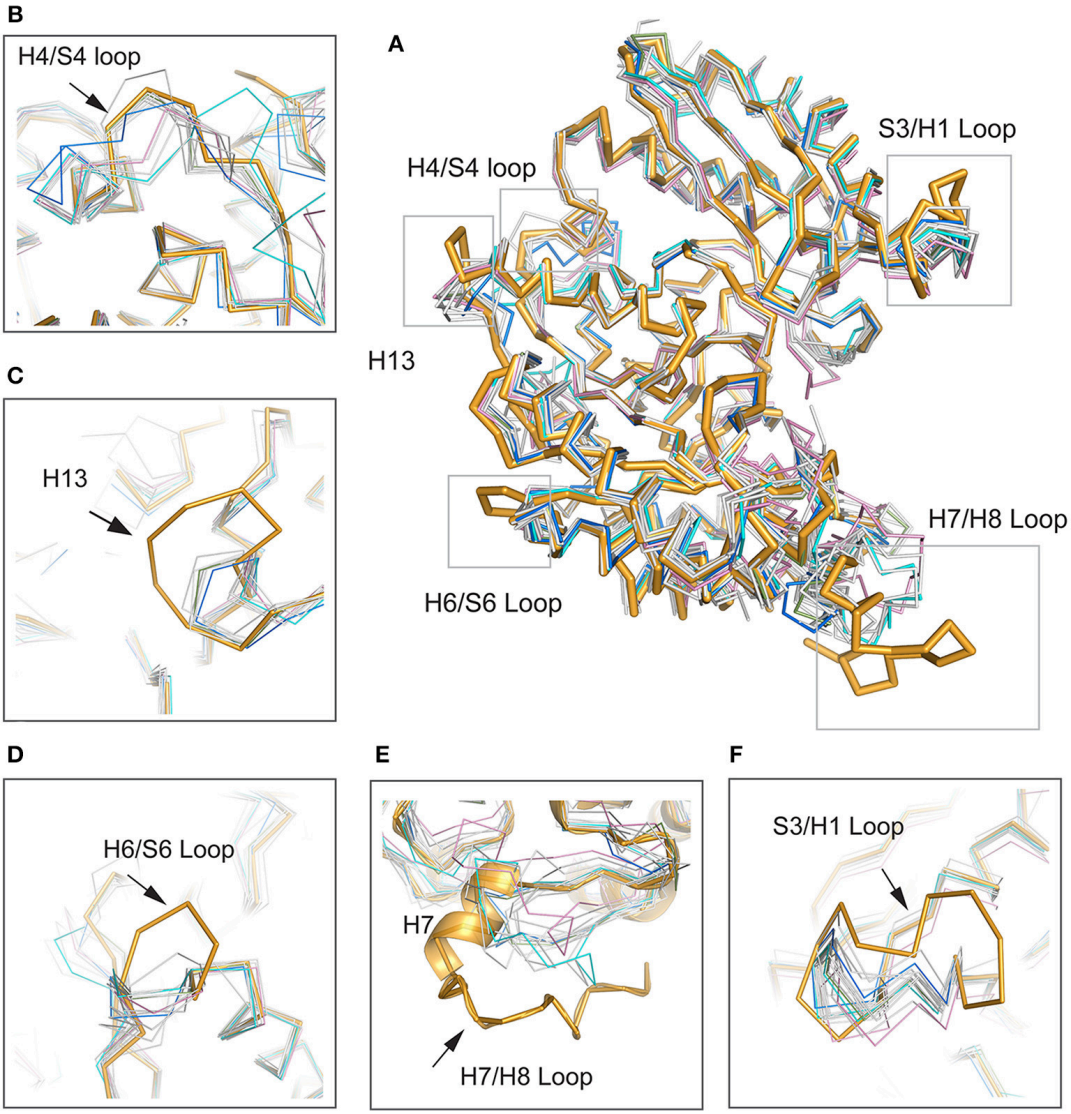
Structural comparisons between *Mhp* Eno and other enolases. *Mhp* Eno is shown in bright orange. Enolases from human, yeast, *E. coli* and *Bacillus subtilis* are shown in pink, cyan, blue and green, respectively, and the other enolases are shown in white. The overall structure is shown in **(A)**. **(B–F)** show enlarged pictures of the H4/S4 loop, H13, H6/S6 loop, H7/H8 loop, and S3/H1 loop regions. H7 is shown in both cartoon and ribbon forms in **(E)**.

To determine the relationships between *Mhp* Eno and other enolases, we compared enolase at both the structural and primary sequence levels (Figures 4, 5). Overall, their similarities coincided with their evolutionary relationships. With the exception of *Mycoplasma* enolases, *Mhp* Eno showed the best identity with enolases of gram-positive bacteria. For example, *Bacillus subtilis* enolase had the most similar structure to that of *Mhp* Eno, with an RMSD of 0.8 Å. In addition, *Enterococcus hirae enolase* had the best sequence identity to *Mhp* Eno (59%). The similarities between *Mhp* Eno and gram-negative bacterial enolases were slightly worse than those of gram-positive bacterial enolases. *Mhp* Eno showed the worst structure and sequence identity with eukaryote enolases. For example, *Mhp* Eno showed only 46% sequence identity with Drosophila enolase and had a 2.0-Å RMSD with human enolase. The superimposition of enolases from different species was performed, and the results clearly showed five sites where *Mhp* Eno showed notable differences from other enolases, particularly the S3/H1 loop, H4/S4 loop, H6/S6 loop, H7/H8 loop, and H13 (Figures 3B, 5). These loops, especially the H7/H8 loop, of *Mhp* Eno were significantly longer than those of other enolases. Until now, with the exception of *Mhp* Eno, such large deviations in the enolase backbone structure have not been observed. All of these results indicated that *M. hyopneumoniae* or *Mycoplasma* has a very unique enolase structure.

### Characteristic Long Loops Are Exposed on the Surface of *Mhp* Eno

In addition to its enzymatic function in the glycolytic pathway, enolase has been implicated in numerous processes, such as DNA-binding and plasmin(ogen) receptor function. These additional functions are more significantly affected by 3D structures and surface properties rather than enzymatic activity (Ehinger et al., 2004; Bruce et al., 2018). To assess the specificity of long loops of *Mhp* Eno, we further compared these areas with those from other enolases. According to the primary sequence comparison, with the exception of *M. hyopneumoniae*, there were 4-5 residue deletions in the S3/H1 loop, 4-6 amino acid deletions in the H6/S6 loop, 8-18 residue deletions in the H7/H8 loop, and 2-5 amino acid deletion in the H13 relative to other enolases. Therefore, these regions were specific to *M. hyopneumoniae* enolase. For the H4/S4 loop, *Mhp* Eno, and most bacterial enolases had ~6 residue deletions, and eukaryote enolases shared a very similar pattern to those of bacterial enolases (Figures 3B, 5A). Therefore, the H4/S4 loop was not an *M. hyopneumoniae-*specific area (Figure 4).

**Figure 6:**
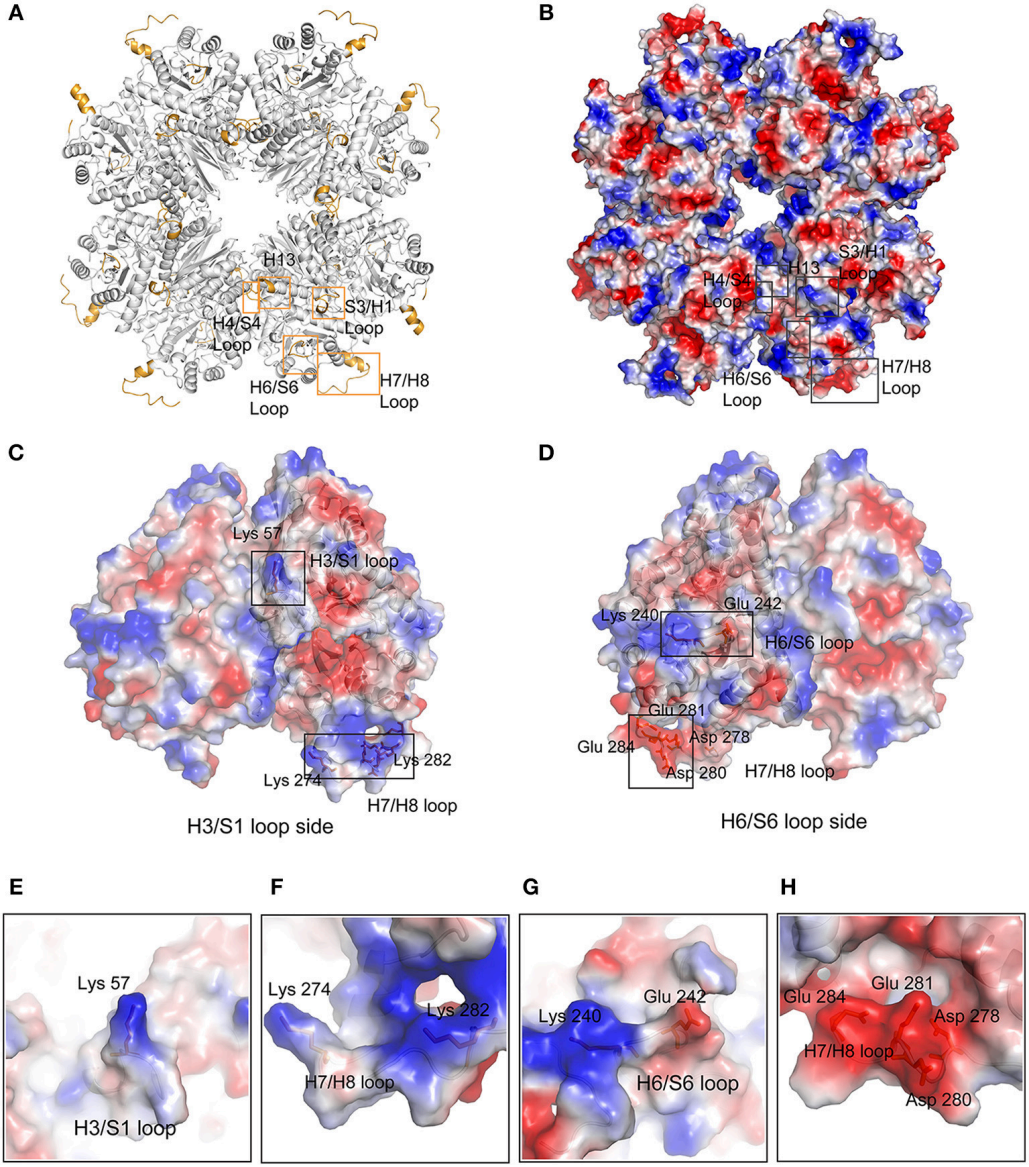
Surface characteristics of featured regions of *Mhp* Eno. **(A)** The featured regions of *Mhp* Eno (bright orange) are shown in cartoon form. **(B)** Electrostatics of the *Mhp* Eno surface. The characteristic regions are boxed by rectangles. The S3/H1 loop region and the H13 region of one enolase molecule are located at the same face as the H6/S6 region of another enolase molecule. **(C)** The H3/S1 loop region side of the dimer unit of *Mhp* Eno. The charged surface residues in the featured regions (Lys57, Lys274, and Lys282) are shown in stick form. **(D)** The H6/S6 loop region side of the dimer unit of *Mhp* Eno. The charged surface residues in the featured regions (Lys240, Lys242, Asp278, Asp280, Glu281, Lys282, and Glu284) are shown in stick form. **(E–H)** The enlarged views of the residues mentioned in **(C,D)**.

However, we do not know the relationship between *Mhp* Eno's characteristic long loops and its adhesion function. It is certain that only loops exposed on the surface can recognize host molecules. Clearly, with the exception of the H4/S4 loop, all regions were located on the surface of *Mhp* Eno (Figures 6A,B). The H4/S4 loop was buried in the dimer interface of *Mhp* Eno. Therefore, it was difficult for the H4/S4 loop to be in contact with other molecules. The H13 region and H6/S6 region were on one side of the disc-like octamer of *Mhp* Eno. The S3/H3 region was located at the opposite side of the octamer (Figures 6C,D). The S3/H3 region, which was on the top region of the disc, was the most accessible region (Figures 6C,E). The H7/H8 loop was at the edge of the octamer and had the largest exposed surface (Figures 6A–D). All of these parameters indicated that the two sides of the disc-like octamer *Mhp* Eno shared the same surface patterns. We further checked the electrostatic potentials of these regions. The H4/S4 and H13 regions showed no notable exposed charged areas. The H6/S6 surface had a positively charged K240 and a negatively charged E242 on (Figures 6D,G). A positively charged K57 was located in the S3/H3 region (Figures 6C,E). This result was interesting for the H7/H8 region because it consisted of a consecutive positively charged patch at 265–274 (KKLKAASLSK) and a negatively charged patch at 275–284 (NQADLDEKFE). However, these two areas were on opposite sides of the *Mhp* Eno disc (Figures 6C,D,F,H).

## Discussion

*Mycoplasma* species are distinguished phenotypically from other bacteria by their minimal size and total lack of cell walls. In taxonomy, the lack of a cell wall is used to separate mycoplasmas from other bacteria in a class named Mollicutes (Razin et al., 1998). To date, various structures of enolases from different species, including human (Kang et al., 2008), *Drosophila melanogaster* (Sun et al., 2017), yeast (Stec and Lebioda, 1990), *E. coli* (Kühnel and Luisi, 2001), *Staphylococcus aureus* (Wu et al., 2015), and *Streptococcus suis* (Lu et al., 2012), have been determined. However, the structure of enolase from Mollicutes, which is one of the smallest self-replicating creatures, has never been solved. Here, the crystal structure of *Mhp* Eno was determined.

Enolase is a highly conserved enzyme throughout evolution both for its structure and its functions. Enolase catalyzes the conversion of 2-phosphoglycerate to phosphoenolpyruvate during both glycolysis and gluconeogenesis in all living beings (Seweryn et al., 2007). The enolase enzyme catalytic sites have two conformations, in which the ions or substrates are bound in a “closed” state or in an apo “open” state (Wu et al., 2015). Here, the *Mhp* Eno structure shows a “closed” state. Compared with the structure of PEP-EnoCa, the structure of *Mhp* Eno has all of the PEP-binding sites at E168, E209, R388, S389, and K359 (Supplementary Figure 3C). All residues are at almost the same sites and have the same conformations as those of PEP-EnoCa. Although we did not determine the PEP-*Mhp* Eno or PAG-*Mhp* Eno structures, structural superimposition indicates that *Mhp* Eno also has conserved enzyme catalytic functions. Furthermore, the enzymatic activity of recombinant *Mhp* Eno was confirmed by an enzymatic assay (Supplementary Figure 2), which indicated that recombinant *Mhp* Eno also has biological activity. Enolase is also involved in the formation of functional *E. coli* RNA degradosomes (Bruce et al., 2018). The complex structure of enolase and RNase E (EBS and AR2 regions) shows that the RNase E segment interacts with the intraprotomer cleft of the enolase dimer (Bruce et al., 2018). The superimposition of *Mhp* Eno on the *E. coli* enolase-RNase E complex showed good consistency with the interaction interface (Supplementary Figure 3D). Thus, *Mhp* Eno might also be included in the RNA metabolism of *M. hyopneumoniae*. These results show that *Mhp* Eno has the conserved functional regions of most enolases.

This study revealed that *Mhp* Eno is a multifunctional protein. This protein not only has conserved sites and structures for 2-phospho-D-glycerate hydrolase catalysis and RNaseE binding (Supplementary Figures 3C,D) but also recognizes fibronectin, factor H and plasminogen (Figure 2C). Mycoplasmas are genome-reduced bacteria that are deficient in many genes involved in metabolic pathways and biosynthesis processes. Amino acids, nucleotides, cholesterol, and other macromolecules need to be supplemented for *Mycoplasma* growth (Minion et al., 2004). To make the existing proteins more functionally efficient, Mycoplasmas have evolved to allow their proteins to have multiple functions. Adherence to mucosal cells is the first and most important step of *M. hyopneumoniae* infection. The use of *M. hyopneumoniae* proteins is maximized for adherence. The present study revealed professional adhesins, such as the P97 and P102 families, as well as large numbers of moonlighting proteins, such as MHJ_0125 (Robinson et al., 2013), MHJ_0461 (Jarocki et al., 2015), EF-Tu (Yu et al., 2018a), FBA (Yu et al., 2018b), *Mhp* Eno. Furthermore, the number of adhesins is enlarged by posttranslation processing (Deutscher et al., 2012). Unlike the limited numbers of receptors of other pathogens, there are various types of molecules for *M. hyopneumoniae* adherence. A promiscuous interaction mode has been found for the adhesion of *M. hyopneumoniae* to its host. In detail, one adhesin of *M. hyopneumoniae* can recognize more than one host target, and one target can also bind to more than one adhesin. The sites for catalytic function and RNA degradosome interaction of *Mhp* Eno are conserved and can be deduced by the complex structures of other enolases. However, the structural characteristics forming the basis of *Mhp* Eno adherence are unknown. Here, the crystal structure of *Mhp* Eno shows the unique characteristics of the *Mycoplasma* enolase structure. In our studies, *Mhp* Eno-specific features, such as the S3/H1 loop, H6/S6 loop, H7/H8 loop, and H13 regions (Figure 5), were found by both sequence alignment and structure comparisons (Figures 4, 5). These regions are located on the surface of the molecule and easily interact with host proteins. Thus, *Mhp* Eno-specific regions should be involved in newly determined Eno functions, such as interactions with fibronectin, factor H and plasminogen. The binding ability of *Mhp* Eno to glycosaminoglycans and actin from the host has not been tested. These regions might also be involved in adherence to glycosaminoglycans, actin or other unrecognized molecules that form the swine respiratory tract epithelium.

Most of the eukaryotic and prokaryotic α-enolases are supposed to bind plasmin(ogen) (Ehinger et al., 2004; Kang et al., 2008). The “FYDKERKVY” loop and two C-terminal lysine residues are believed to bind plasminogen in *S. pneumoniae* (Ehinger et al., 2004). In *Mhp* Eno, the C-terminal double lysine residues are conserved. The “FYDKERKVY” motifs are partially found in *Mhp* Eno at “FYNGQKY” sites. We mapped these regions on the octameric *Mhp* Eno. The “FYNGQKY” loop is located on the *Mhp* Eno surface and next to the H7/H8 loop (Supplementary Figures 3A,B). The C-terminal tail is found in the dimer interface, as observed in other enolases (Supplementary Figure 3B). However, according to the sequence alignment, both plasminogen-binding regions (the loop region and the C-terminal lysine residues) are not strictly conserved throughout evolution (Figure 4). Thus, enolases from different species might use different regions to recognize plasminogen. A salt bridge is one of the strongest noncovalent interactions between two proteins. For *S. pneumoniae* enolase, the charged amino acids D250, E252, K251, and K254 in the “FYDKERKVY” loop are critical for plasmin(ogen) binding(Ehinger et al., 2004). It is interesting to find that many charged sites exist in the featured loop regions, such as K240 and E242 in the H6/S6 loop, K57 in the S3/H3 region and a positively charged patch and a negatively charged patch in the H7/H8 loop. Furthermore, these sites are exposed on the surface of *Mhp* Eno, making them easily accessible to plasminogen. Thus, these sites are possibly involved in the interaction with plasminogen. However, an enolase-plasminogen complex structure is not currently available for determining detailed interaction features.

Because plasminogen is a common receptor of enolase (Bergmann et al., 2001; Ehinger et al., 2004; Seweryn et al., 2007; Agarwal et al., 2008), its activation triggers its conversion to plasmin, which is a serine protease that plays important roles in the maintenance of vascular patency and cell migration (Plow et al., 1995). In recent years, the mechanism through which human enolase induces tumor cell invasion and metastasis has been thought to be related to the activation of plasminogen (Godier and Hunt, 2013; Viedma-Rodríguez et al., 2018). For pathogens, host plasminogen is activated by enolase to function in the processes of tissue remodeling, extracellular matrix degradation and bacterial invasion (Bergmann et al., 2001, 2013; Agarwal et al., 2008; Feng et al., 2009). In *M. hyopneumoniae* infection, ciliostasis and the loss of cilia are thought to be the result of the activation of various proteases and amino peptidases, including plasminogen (Robinson et al., 2013). In some studies, *M. hyopneumoniae* was also detected in some nonrespiratory system organs, such as the liver and kidney (Razin et al., 1998). This result might be related to plasminogen activation by *M. hyopneumoniae* surface proteins, such as enolase. Additionally, in our previous studies, *Mhp* Eno was found to be an important virulence factor candidate whose expression level was significantly increased after infection. In this study, *Mhp* Eno showed strong binding to plasminogen, as verified by far-WB and SPR. This result indicated that *Mhp* Eno is involved in *M. hyopneumoniae* pathogenicity, which is induced by host plasminogen activation.

In conclusion, we have reported the first enolase structure from a *Mycoplasma* species. Structural and sequence analyses revealed that *Mhp* Eno has four characteristic regions that are different from those of other enolases. *Mhp* Eno is a cell-surface protein of *M. hyopneumoniae* and induces adhesion to STECs. In addition, *Mhp* Eno is a multifunction protein that not only has enzyme catalytic sites and RNase E-binding regions but also recognizes fibronectin, factor H and plasminogen. Our study will aid the investigation of enolase evolution and provide insights into the pathogenesis of *M. hyopneumoniae*.

## Data Availability

The coordinates and structural factors generated in this study were submitted to the Protein Data Bank under the following accession number: https://www.rcsb.org/structure/6J36 (Supplementary Datasheet 2).

## Author Contributions

RC, YY, RG, XX, ZZ, WW, and TR performed the experimental work. RC, YY, ZF, WW, QinX, WZ, QiyX, and GS performed the data analyses, and all research was conducted under the supervision of WW, WZ, and GS. The original draft of the manuscript was written by RC and YY, and revisions were made by TR, WZ, and GS.

### Conflict of Interest Statement

The authors declare that the research was conducted in the absence of any commercial or financial relationships that could be construed as a potential conflict of interest.

## References

[B1] Adams C. Pitzer J. Minion F. C. (2005). *In vivo* expression analysis of the P97 and P102 paralog families of *Mycoplasma hyopneumoniae*. Infect. Immun. 73, 7784–7787. 10.1128/IAI.73.11.7784-7787.2005 16239586 PMC1273896

[B2] Agarwal S. Kulshreshtha P. Bambah Mukku D. Bhatnagar R. (2008). alpha-Enolase binds to human plasminogen on the surface of *Bacillus anthracis*. Biochim. Biophys. Acta 1784, 986–994. 10.1016/j.bbapap.2008.03.017 18456007

[B3] Bergmann S. Rohde M. Chhatwal G. S. Hammerschmidt S. (2001). alpha-Enolase of *Streptococcus pneumoniae* is a plasmin(ogen)-binding protein displayed on the bacterial cell surface. Mol. Microbiol. 40, 1273–1287. 10.1046/j.1365-2958.2001.02448.x 11442827

[B4] Bergmann S. Schoenen H. Hammerschmidt S. (2013). The interaction between bacterial enolase and plasminogen promotes adherence of *Streptococcus pneumoniae* to epithelial and endothelial cells. Int. J. Med. Microbiol. 303, 452–462. 10.1016/j.ijmm.2013.06.002 23906818

[B5] Blanchard B. Vena M. M. Cavalier A. Le Lannic J. Gouranton J. Kobisch M. (1992). Electron microscopic observation of the respiratory tract of SPF piglets inoculated with *Mycoplasma hyopneumoniae*. Vet. Microbiol. 30, 329–341. 10.1016/0378-1135(92)90020-T 1533978

[B6] Brown C. K. Kuhlman P. L. Mattingly S. Slates K. Calie P. J. Farrar W. W. (1998). A model of the quaternary structure of enolases, based on structural and evolutionary analysis of the octameric enolase from *Bacillus subtilis*. J. Protein Chem. 17, 855–866. 10.1023/A:1020790604887 9988532

[B7] Bruce H. A. Du D. Matak-Vinkovic D. Bandyra K. J. Broadhurst R. W. Martin E. (2018). Analysis of the natively unstructured RNA/protein-recognition core in the *Escherichia coli* RNA degradosome and its interactions with regulatory RNA/Hfq complexes. Nucleic Acids Res. 46, 387–402. 10.1093/nar/gkx1083 29136196 PMC5758883

[B8] DeBey M. C. Ross R. F. (1994). Ciliostasis and loss of cilia induced by *Mycoplasma hyopneumoniae* in porcine tracheal organ cultures. Infect. Immun. 62, 5312–5318. 7960110 10.1128/iai.62.12.5312-5318.1994 PMC303270

[B9] Deutscher A. T. Jenkins C. Minion F. C. Seymour L. M. Padula M. P. Dixon N. E. (2010). Repeat regions R1 and R2 in the P97 paralogue Mhp271 of *Mycoplasma hyopneumoniae* bind heparin, fibronectin and porcine cilia. Mol. Microbiol. 78, 444–458. 10.1111/j.1365-2958.2010.07345.x 20879998

[B10] Deutscher A. T. Tacchi J. L. Minion F. C. Padula M. P. Crossett B. Bogema D. R. (2012). *Mycoplasma hyopneumoniae* surface proteins Mhp385 and Mhp384 bind host cilia and glycosaminoglycans and are endoproteolytically processed by proteases that recognize different cleavage motifs. J. Proteome Res. 11, 1924–1936. 10.1021/pr201115v 22229926

[B11] Díaz-Ramos A. Roig-Borrellas A. García-Melero A. López-Alemany R. (2012). alpha-Enolase, a multifunctional protein: its role on pathophysiological situations. J. Biomed. Biotechnol. 2012:156795 10.1155/2012/156795 23118496 PMC3479624

[B12] DiMaio F. Echols N. Headd J. J. Terwilliger T. C. Adams P. D. Baker D. (2013). Improved low-resolution crystallographic refinement with Phenix and Rosetta. Nat. Methods 10, 1102–1104. 10.1038/nmeth.2648 24076763 PMC4116791

[B13] Ehinger S. Schubert W. D. Bergmann S. Hammerschmidt S. Heinz D. W. (2004). Plasmin(ogen)-binding alpha-enolase from *Streptococcus pneumoniae*: crystal structure and evaluation of plasmin(ogen)-binding sites. J. Mol. Biol. 343, 997–1005. 10.1016/j.jmb.2004.08.088 15476816

[B14] Emsley P. Cowtan K. (2004). Coot: model-building tools for molecular graphics. Acta Crystallogr. Section D Biol. Crystallogr. 60, 2126–2132. 10.1107/S0907444904019158 15572765

[B15] Feng Y. Pan X. Sun W. Wang C. Zhang H. Li X. Tang J. (2009). *Streptococcus suis* enolase functions as a protective antigen displayed on the bacterial cell surface. J. Infect. Dis. 200, 1583–1592. 10.1086/644602 19848587

[B16] Garcia-Morante B. Segalés J. Fraile L. Pérez de Rozas A. Maiti H. Coll T. (2016). Assessment of *Mycoplasma hyopneumoniae*-induced pneumonia using different lung lesion scoring systems: a comparative review. J. Comp. Pathol. 154, 125–134. 10.1016/j.jcpa.2015.11.003 26774274

[B17] Gibson D. G. Glass J. I. Lartigue C. Noskov V. N. Chuang R. Y. Algire M. A. (2010). Creation of a bacterial cell controlled by a chemically synthesized genome. Science 329, 52–56. 10.1126/science.1190719 20488990

[B18] Glass J. I. Assad-Garcia N. Alperovich N. Yooseph S. Lewis M. R. Maruf M. (2006). Essential genes of a minimal bacterium. Proc. Natl. Acad. Sci. U.S.A. 103, 425–430. 10.1073/pnas.0510013103 16407165 PMC1324956

[B19] Godier A. Hunt B. J. (2013). Plasminogen receptors and their role in the pathogenesis of inflammatory, autoimmune and malignant disease. J. Thromb. Haemost. 11, 26–34. 10.1111/jth.12064 23140188

[B20] Hough M. A. Wilson K. S. (2018). From crystal to structure with CCP4. Acta Crystallogr. Section D Struct. Biol. 74:67 10.1107/S2059798317017557 29533232 PMC5947770

[B21] Hsu T. Minion F. C. (1998). Identification of the cilium binding epitope of the *Mycoplasma hyopneumoniae* P97 adhesin. Infect. Immun. 66, 4762–4766. 9746576 10.1128/iai.66.10.4762-4766.1998 PMC108587

[B22] Hutchison C. A. III. Chuang R. Y. Noskov V. N. Assad-Garcia N. Deerinck T. J. Ellisman M. H. (2016). Design and synthesis of a minimal bacterial genome. Science 351:aad6253 10.1126/science.aad6253 27013737

[B23] Jarocki V. M. Santos J. Tacchi J. L. Raymond B. B. Deutscher A. T. Jenkins C. (2015). MHJ_0461 is a multifunctional leucine aminopeptidase on the surface of *Mycoplasma hyopneumoniae*. Open Biol. 5:140175 10.1098/rsob.140175 25589579 PMC4313372

[B24] Jenkins C. Wilton J. L. Minion F. C. Falconer L. Walker M. J. Djordjevic S. P. (2006). Two domains within the *Mycoplasma hyopneumoniae* cilium adhesin bind heparin. Infect. Immun. 74, 481–487. 10.1128/IAI.74.1.481-487.2006 16369004 PMC1346629

[B25] Kang H. J. Jung S. K. Kim S. J. Chung S. J. (2008). Structure of human alpha-enolase (hENO1), a multifunctional glycolytic enzyme. Acta Crystallogr. Section D Biol. Crystallogr. 64, 651–657. 10.1107/S0907444908008561 18560153

[B26] Kovalevskiy O. Nicholls R. A. Long F. Carlon A. Murshudov G. N. (2018). Overview of refinement procedures within REFMAC5: utilizing data from different sources. Acta Crystallogr. Section D Struct. Biol. 74, 215–227. 10.1107/S2059798318000979 29533229 PMC5947762

[B27] Kühnel K. Luisi B. F. (2001). Crystal structure of the *Escherichia coli* RNA degradosome component enolase. J. Mol. Biol. 313, 583–592. 10.1006/jmbi.2001.5065 11676541

[B28] Kureljusic B. Weissenbacher-Lang C. Nedorost N. Stixenberger D. Weissenbock H. (2016). Association between Pneumocystis spp. and co-infections with *Bordetella bronchiseptica, Mycoplasma hyopneumoniae and Pasteurella multocida in Austrian pigs with pneumonia*. Vet. J. 207, 177–179. 10.1016/j.tvjl.2015.11.003 26654847

[B29] Li B. Du L. Xu X. Sun B. Yu Z. Feng Z. (2015a). Transcription analysis on response of porcine alveolar macrophages to co-infection of the highly pathogenic porcine reproductive and respiratory syndrome virus and *Mycoplasma hyopneumoniae*. Virus Res. 196, 60–69. 10.1016/j.virusres.2014.11.006 25445346

[B30] Li Q. Liu H. Du D. Yu Y. Ma C. Jiao F. (2015b). Identification of novel laminin- and fibronectin-binding proteins by far-western blot: capturing the adhesins of *Streptococcus suis* Type 2. Front. Cell. Infect. Microbiol. 5:82 10.3389/fcimb.2015.00082 26636044 PMC4644805

[B31] Liu W. Xiao S. Li M. Guo S. Li S. Luo R. (2013). Comparative genomic analyses of Mycoplasma hyopneumoniae pathogenic 168 strain and its high-passaged attenuated strain. BMC Genomics 14:80 10.1186/1471-2164-14-80 23384176 PMC3626624

[B32] Lu Q. Lu H. Qi J. Lu G. Gao G. F. (2012). An octamer of enolase from *Streptococcus suis*. Protein Cell 3, 769–780. 10.1007/s13238-012-2040-7 23055041 PMC4875344

[B33] Maes D. Sibila M. Kuhnert P. Segalés J. Haesebrouck F. Pieters M. (2017). Update on *Mycoplasma hyopneumoniae* infections in pigs: knowledge gaps for improved disease control. Transbound Emerg. Dis. 65(Suppl. 1), 110–124. 10.1111/tbed.12677 28834294

[B34] Maes D. Verdonck M. Deluyker H. de Kruif A. (1996). Enzootic pneumonia in pigs. Vet. Q. 18, 104–109. 10.1080/01652176.1996.9694628 8903144

[B35] Minion F. C. Adams C. Hsu T. (2000). R1 region of P97 mediates adherence of *Mycoplasma hyopneumoniae* to swine cilia. Infect. Immun. 68, 3056–3060. 10.1128/IAI.68.5.3056-3060.2000 10769015 PMC97530

[B36] Minion F. C. Lefkowitz E. J. Madsen M. L. Cleary B. J. Swartzell S. M. Mahairas G. G. (2004). The genome sequence of *Mycoplasma hyopneumoniae* strain 232, the agent of swine mycoplasmosis. J. Bacteriol. 186, 7123–7133. 10.1128/JB.186.21.7123-7133.2004 15489423 PMC523201

[B37] Newman J. A. Hewitt L. Rodrigues C. Solovyova A. S. Harwood C. R. Lewis R. J. (2012). Dissection of the network of interactions that links RNA processing with glycolysis in the *Bacillus subtilis* degradosome. J. Mol. Biol. 416, 121–136. 10.1016/j.jmb.2011.12.024 22198292

[B38] Plow E. F. Herren T. Redlitz A. Miles L. A. Hoover-Plow J. L. (1995). The cell biology of the plasminogen system. FASEB J. 9, 939–945. 10.1096/fasebj.9.10.7615163 7615163

[B39] Raymond B. B. Tacchi J. L. Jarocki V. M. Minion F. C. Padula M. P. Djordjevic S. P. (2013). P159 from *Mycoplasma hyopneumoniae* binds porcine cilia and heparin and is cleaved in a manner akin to ectodomain shedding. J. Proteome Res. 12, 5891–5903. 10.1021/pr400903s 24195521

[B40] Raymond B. B. A. Madhkoor R. Schleicher I. Uphoff C. C. Turnbull L. Whitchurch C. B. (2018). Extracellular actin is a receptor for *Mycoplasma hyopneumoniae*. Front. Cell. Infect. Microbiol. 8:54 10.3389/fcimb.2018.00054 29535975 PMC5835332

[B41] Razin S. Yogev D. Naot Y. (1998). Molecular biology and pathogenicity of mycoplasmas. Microbiol. Mol. Biol. Rev. 62, 1094–1156. 9841667 10.1128/mmbr.62.4.1094-1156.1998 PMC98941

[B42] Reed G. H. Poyner R. R. Larsen T. M. Wedekind J. E. Rayment I. (1996). Structural and mechanistic studies of enolase. Curr. Opin. Struct. Biol. 6, 736–743. 10.1016/S0959-440X(96)80002-9 8994873

[B43] Robinson M. W. Buchtmann K. A. Jenkins C. Tacchi J. L. Raymond B. B. To J. (2013). MHJ_0125 is an M42 glutamyl aminopeptidase that moonlights as a multifunctional adhesin on the surface of *Mycoplasma hyopneumoniae*. Open Biol. 3:130017 10.1098/rsob.130017 23594879 PMC3718333

[B44] Seweryn E. Pietkiewicz J. Szamborska A. Gamian A. (2007). [Enolase on the surface of prockaryotic and eukaryotic cells is a receptor for human plasminogen]. Postepy Hig. Med. Dosw. 61, 672–682. 18033204

[B45] Sibila M. Pieters M. Molitor T. Maes D. Haesebrouck F. Segalés J. (2009). Current perspectives on the diagnosis and epidemiology of *Mycoplasma hyopneumoniae* infection. Vet. J. 181, 221–231. 10.1016/j.tvjl.2008.02.020 18396428 PMC7110805

[B46] Siqueira F. M. Thompson C. E. Virginio V. G. Gonchoroski T. Reolon L. Almeida L. G. (2013). New insights on the biology of swine respiratory tract mycoplasmas from a comparative genome analysis. BMC Genomics 14:175 10.1186/1471-2164-14-175 23497205 PMC3610235

[B47] Sladek T. L. (1986). A hypothesis for the mechanism of mycoplasma evolution. J. Theor. Biol. 120, 457–465. 10.1016/S0022-5193(86)80039-X 3795988

[B48] Stec B. Lebioda L. (1990). Refined structure of yeast apo-enolase at 2.25 A resolution. J. Mol. Biol. 211, 235–248. 10.1016/0022-2836(90)90023-F 2405163

[B49] Sun C. Xu B. Liu X. Zhang Z. Su Z. (2017). Crystal structure of enolase from *Drosophila melanogaster*. Acta Crystallogr. Section F Struct. Biol. Commun. 73, 228–234. 10.1107/S2053230X17004022 28368282 PMC5379173

[B50] Tacchi J. L. Raymond B. B. Haynes P. A. Berry I. J. Widjaja M. Bogema D. R. (2016). Post-translational processing targets functionally diverse proteins in *Mycoplasma hyopneumoniae*. Open Biol. 6:150210 10.1098/rsob.150210 26865024 PMC4772806

[B51] Viedma-Rodríguez R. Martínez-Hernández M. G. Flores-López L. A. Baiza-Gutman L. A. (2018). Epsilon-aminocaproic acid prevents high glucose and insulin induced-invasiveness in MDA-MB-231 breast cancer cells, modulating the plasminogen activator system. Mol. Cell. Biochem. 437, 65–80. 10.1007/s11010-017-3096-8 28612231

[B52] Wold F. Ballou C. E. (1957a). Studies on the enzyme enolase. II. Kinetic studies. J. Biol. Chem. 227, 313–328. 13449075

[B53] Wold F. Ballou C. E. (1957b). Studies on the enzyme enolase. I. Equilibrium studies. J. Biol. Chem. 227, 301–312. 13449074

[B54] Wu Y. Wang C. Lin S. Wu M. Han L. Tian C. (2015). Octameric structure of *Staphylococcus aureus* enolase in complex with phosphoenolpyruvate. Acta Crystallogr. Section DBiol. Crystallogr. 71, 2457–2470. 10.1107/S1399004715018830 PMC4667285 26627653

[B55] Yu Y. Liu M. Hua L. Qiu M. Zhang W. Wei Y. (2018b). Fructose-1,6-bisphosphate aldolase encoded by a core gene of *Mycoplasma hyopneumoniae* contributes to host cell adhesion. Vet. Res. 49:114 10.1186/s13567-018-0610-2 30454073 PMC6245935

[B56] Yu Y. Wang H. Wang J. Feng Z. Wu M. Liu B. (2018a). Elongation factor thermo unstable (EF-Tu) moonlights as an adhesin on the surface of *Mycoplasma hyopneumoniae* by binding to fibronectin. Front. Microbiol. 9:974 10.3389/fmicb.2018.00974 29867877 PMC5962738

