# Supplementary material for: Featured Species-Specific Loops Are Found in the Crystal Structure of Mhp Eno, a Cell Surface Adhesin From Mycoplasma hyopneumoniae
Source: Front Cell Infect Microbiol. 2019 Jun 13;9:209. doi: 10.3389/fcimb.2019.00209 (PMC6585157; doi:10.3389/fcimb.2019.00209)
Supplement: Supplementary file 1 [file Data_Sheet_1.pdf]

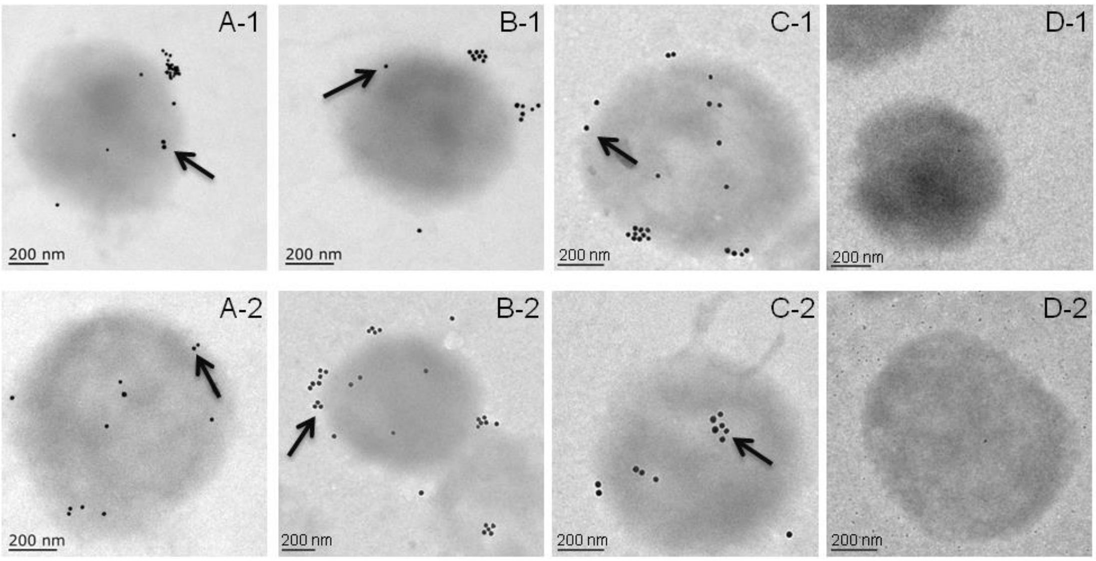

Supplementary figure 1| Detection of *Mhp* Eno on the surface of *M. hyopneumoniae* by immune electron microscopy. (A1-2) *M. hyopneumoniae* strain 168 treated with anti-*Mhp* Eno serum and secondary gold-conjugated antibodies. (B1-2) *M. hyopneumoniae* strain NJ treated with anti-*Mhp* Eno serum and secondary gold-conjugated antibodies. (C1-2) *M. hyopneumoniae* strain WX treated with anti-*Mhp* Eno serum and secondary gold-conjugated antibodies. (D1-2) *M. hyopneumoniae* strain treated with preimmune serum and secondary gold-conjugated antibodies.

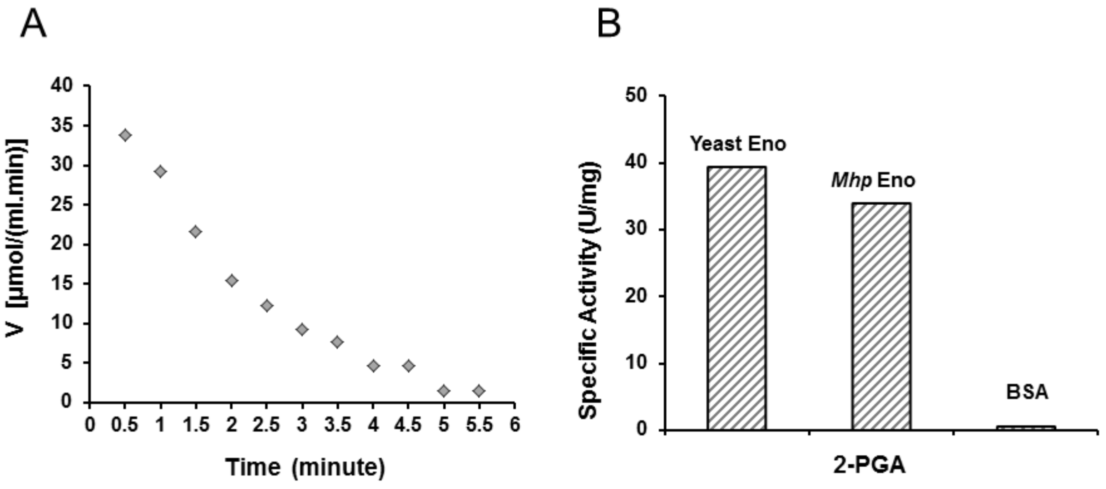

Supplementary figure 2| Enzyme activity of *Mhp* Eno for the catalysis of the conversion of 2-PGA to PEP. (A) Velocity of the enzyme-catalyzed reaction of *Mhp* Eno. The assay was measured at 0.5-min intervals for 5.5 min. (B) Specific activity of *Mhp* Eno. The specific activities of yeast Eno (as a positive control), *Mhp* Eno and BSA (as a negative control) for the conversion of 2-PGA to PEP were determined.

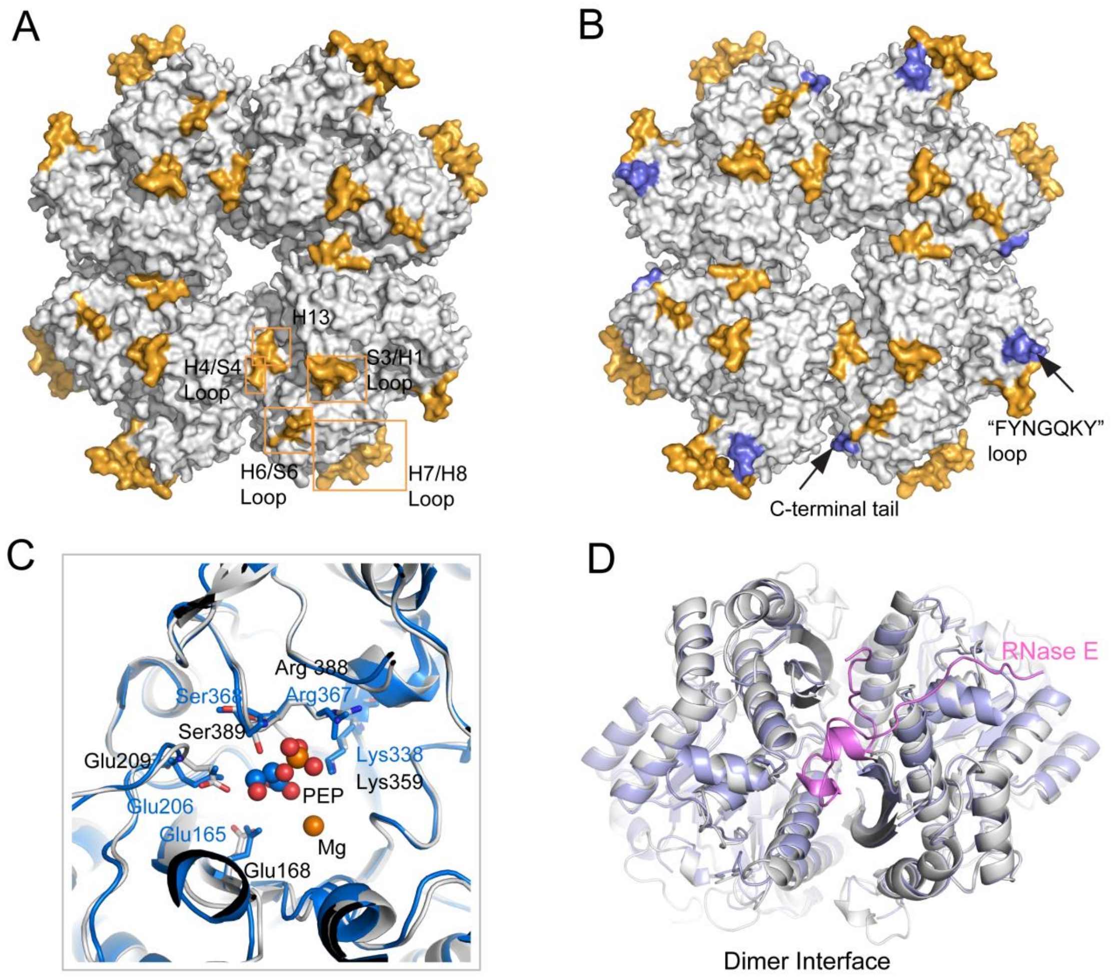

Supplementary figure 3 | Conserved sites of *Mhp* Eno for traditional enolase functions. (A) The featured regions of *Mhp* Eno (bright orange) are shown in surface form. (B) Hypothesized plasminogen-binding regions in octameric *Mhp* Eno. The "FYNGQKY" loops and "KK" C-terminal tail are shown in slate. (C) Superimposition between the *Mhp* Eno structure and the enolase-PEP complex. The enolase-PEP complex (PDB ID: 5BOF) is from *Staphylococcus aureus* and is shown in blue. The residues for PEP binding are shown as blue sticks. The coordinated sites of *Mhp* Eno are shown as white sticks. (D) Superimposition between the *Mhp* Eno and the RNase E-enolase complex structure. *Mhp* Eno is shown in white. The RNase E-enolase complex is from *E. coli* (PDB ID: 5OHG). RNase E is shown in pink, and *E. coli* enolase is shown in light slate.

Supplementary Table 1. The Dimmer Interface.

Molecule A	Molecule B
Hydrogen bonds	
Arg 10 $N^{\eta1}$	Glu 431 $O^{\varepsilon2}$
Leu 13N , Leu 13 O	Asn 424 $O^{\delta1}$, Asn 424 $N^{\delta2}$
Ser 15 O	Arg 416 N
Gly 17 O , Gly 17 O	His 191 $N^{\varepsilon2}$, Asn 187 $N^{\delta2}$
Asp 60 O	Lys 188 N^{ζ}
Tyr 63 N	Asn 187 $O^{\delta1}$
Ser 161 O^{γ}	Glu 203 $O^{\varepsilon1}$
Lys 180 N^{ζ}	Glu 11 O , Glu11 $O^{\varepsilon1}$
Asn 187 $O^{\delta1}$, Asn 187 $N^{\delta2}$	Tyr 63 N , Gly17 O
Lys 188 N^{ζ}	Asp 60 O
His 191 $N^{\varepsilon2}$	Asn18 $O^{\delta1}$, Gly17 O
Glu 203$O^{\varepsilon1}$	Ser 161 O^{γ}
Gln 205 $O^{\varepsilon1}$	Ala 213 N ,Gly 207 N
Val 206 N ,Val 206 O	Val 206 O , Val 206 N
Gly 207 N	Gln 205 $O^{\varepsilon1}$
Ala 213 N , Ala 213 O	Gln 205 $O^{\varepsilon1}$, Gln 205 $N^{\varepsilon2}$
Glu 391 O	Thr 417 $O^{\gamma1}$
Arg 416 N	Ser15 O
Thr 417 N, Thr 417 $O^{\gamma1}$	Asp 418 $O^{\delta1}$
Asp 418 $O^{\delta1}$	Thr 417 N
Asn 424 $O^{\delta1}$, Asn 424 $N^{\delta2}$	Leu 13 N , Leu 13 O
Glu 431 $O^{\varepsilon1}$, Glu 431 $O^{\varepsilon2}$	Arg 10 N^{ε}, Arg 10 $N^{\eta2}$
Salt bridges	
Arg 10 $N^{\eta1}$	Glu 431 $O^{\varepsilon1}$, Glu 431 $O^{\varepsilon2}$
Lys 180 N^{ζ}	Lys 180 N^{ζ}
Glu 431 $O^{\varepsilon1}$, Glu 431 $O^{\varepsilon2}$	Arg 10 N^{ε} , Arg 10 $N^{\eta2}$
Van der Waals Contacts	
Lys 6, Phe 8, Glu 11, Ile 12, Asp 14, Arg 16, Asn 18, Pro 19, Gln 22, Asn 61, Trp 62, Lys 66, Met 69, His 159, Leu 183, Gln 184, Phe 190, Asn 192, Lys 195, Lys 198, Thr 204, Phe 212, Ser 392, Glu 393, Thr 395, Leu 414, Ser 415, Ile 420, Ala 421, Tyr 423, Leu 427, Val 428, Tyr 436	Lys 6, Phe 8, Ile 12, Asp 14, Arg 16, Pro 19, Gln 22, Asn 61, Trp 62, Lys 66, Met 69, His 159, Leu 183, Gln 184, Phe 190, Asn 192, Lys 195, Lys 198, Thr 204, Gly 211, Asn 215, Ser 392, Glu 393, Thr 395, Leu 414, Ser 415, Ile 420, Ala 421, Tyr 423, Leu 427, Val 428, Tyr 436

Supplementary Table 2 The Octamer Interface.

Molecule A	Molecule B
Hydrogen bonds	
Arg 136 $N^{\eta 1}$, Arg 136 $N^{\eta 2}$	Lys 89 O, Tyr 137 O^{η}
Thr 141 N	Tyr 137 O, Leu 138
Arg 92 $N^{\eta 1}$	Asn 142 $O^{\delta 1}$
Asn 142 $N^{\delta 2}$	Asp 371 $O^{\delta 1}$
Leu 93 N	Tyr 436 O
Lys 89 O	Arg 136 $N^{\eta 2}$, Arg 136 $N^{\eta 1}$
Tyr 137 O, Tyr 137 O^{η}	Thr 141 N, Arg 136 $N^{\eta 2}$
Leu 138 O	Thr 141 N
Asn 142 $O^{\delta 1}$	Arg 92 $N^{\eta 1}$
Asp 371 $O^{\delta 1}$	Asn 142 $N^{\delta 2}$
Tyr 436 O	Leu 93 N
Van der Waals Contacts	
Ser 2, Cys 87, Asn 90, Gln 91, Gln 96, Glu 129, Leu 130, Arg 131, Met 132, Pro 133, Gly 139, Gly 140, Pro 143, Thr 144, Leu 145, Lys 351, Leu 366, Ser 367, Leu 370, His 374, Gln 377, Lys 378, Gly 380, Ser 405, Tyr 432, Ser 435, Ala 437, Lys 438, Ile 442, Glu 445, Tyr 448	Ser 2, Asn 90, Gln 91, Leu 130, Arg 131, Met 132, Gly 139, Gly 140, Pro 143, Thr 144, Leu 145, Leu 366, Ser 367, Leu 370, His 374, Gln 377, Lys 378, Gly 380, Val 404, Ser 405, Tyr 432, Ser 435, Ala 437, Lys 438, Ile 442, Glu 445, Tyr 448
